# Molecular mechanisms and therapeutic target of NETosis in diseases

**DOI:** 10.1002/mco2.162

**Published:** 2022-08-19

**Authors:** Jiayu Huang, Weiqi Hong, Meihua Wan, Limin Zheng

**Affiliations:** ^1^ Laboratory of Aging Research and Cancer Drug Target State Key Laboratory of Biotherapy National Clinical Research Center for Geriatrics West China Hospital Sichuan University Chengdu China; ^2^ Department of Integrated Traditional Chinese and Western Medicine West China Hospital Sichuan University Chengdu Sichuan China; ^3^ Guangdong Province Key Laboratory of Pharmaceutical Functional Genes MOE Key Laboratory of Gene Function and Regulation School of Life Sciences Sun Yat‐Sen University Guangzhou China; ^4^ State Key Laboratory of Oncology in Southern China Collaborative Innovation Center for Cancer Medicine Sun Yat‐Sen University Cancer Center Guangzhou China

**Keywords:** NET inhibition, NETosis, neutrophil, neutrophil extracellular trap

## Abstract

Evidence shows that neutrophils can protect the host against pathogens in multiple ways, including the formation and release of neutrophil extracellular traps (NETs). NETs are web‐like structures composed of fibers, DNA, histones, and various neutrophil granule proteins. NETs can capture and kill pathogens, including bacteria, viruses, fungi, and protozoa. The process of NET formation is called NETosis. According to whether they depend on nicotinamide adenine dinucleotide phosphate (NADPH), NETosis can be divided into two categories: “suicidal” NETosis and “vital” NETosis. However, NET components, including neutrophil elastase, myeloperoxidase, and cell‐free DNA, cause a proinflammatory response and potentially severe diseases. Compelling evidence indicates a link between NETs and the pathogenesis of a number of diseases, including sepsis, systemic lupus erythematosus, rheumatoid arthritis, small‐vessel vasculitis, inflammatory bowel disease, cancer, COVID‐19, and others. Therefore, targeting the process and products of NETosis is critical for treating diseases linked with NETosis. Researchers have discovered that several NET inhibitors, such as toll‐like receptor inhibitors and reactive oxygen species scavengers, can prevent uncontrolled NET development. This review summarizes the mechanism of NETosis, the receptors associated with NETosis, the pathology of NETosis‐induced diseases, and NETosis‐targeted therapy.

## Graphical abstract

NETs are involved in the pathogenesis and progression of various diseases, such as sepsis, SLE, RA, SVV, IBD, cancer, and COVID‐19. Components of NETs may act as autoantigens, leading to inflammation and autoimmune diseases. In addition, some diseases aggravate NETosis and cause a vicious circle.

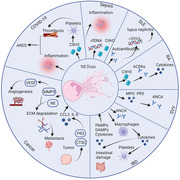

## 1 INTRODUCTION

Neutrophils are the most common responsive innate immune effector cells and the first line of defense against pathogen invasion through degranulation, phagocytosis, reactive oxygen species (ROS) generation, and chemokine and cytokine synthesis.^1^, ^2^, ^3^ Furthermore, scientists have discovered another critical mechanism by which neutrophils protect the host from infections. Brinkmann et al.^4^ reported that neutrophils stimulated with phorbol 12‐myristate 13‐acetate (PMA) could form web‐like structures termed neutrophil extracellular traps (NETs) that could be induced by both extracellular or intracellular pathogens, which were capable of capturing and killing bacteria, viruses,^5^ fungi,^6^ and protozoa.^7^ NETs are made up of fibers, DNA, histones, and various neutrophil granule proteins, including neutrophil elastase (NE), cathelicidin, cathepsin G, and myeloperoxidase (MPO).^8^, ^9^ The ensuing programmed cell death is referred to as NETosis, and it is classified into two types: “suicidal” NETosis and “vital” NETosis.^10^ The neutrophil nucleus loses its shape and chromatin decondensation during NETosis. The membrane and nucleus particles then disintegrate and mix together, releasing them to the outside of the cell.^11^ Apart from neutrophils, other immune cells, such as basophils,^12^ eosinophils,^13^ mast cells,^14^ and macrophages,^15^ may also generate this web‐like structure.

NETosis is a double‐edged sword for the immune system. NETs include a variety of antimicrobial proteins, including antimicrobial peptide‐LL37, pentraxin 3, proteinase 3 (PR3), lactoferrin, MPO, and others.^16^, ^17^, ^18^ Compelling evidence has shown that neutrophils play a significant role in the immune response and that NETs can encapsulate, capture, and kill pathogens, particularly large microorganisms such as *Candida albicans* and *Mycobacterium bovis* aggregates that are difficult to swallow.^19^ Furthermore, once viruses activate neutrophils, NETs are produced and are capable of trapping and eliminating viruses or repressing viral proliferation by blocking the protein kinase C (PKC) pathway.^20^, ^21^, ^22^

However, excessive release or dysfunction of NETs can trigger and amplify inflammatory responses, which can cause tissue damage and a variety of diseases. The components of NETs might become autoantigens, causing inflammation and autoimmune diseases. B lymphocytes, for example, may develop autoantibodies against NET‐derived cell‐free DNA (cfDNA) in individuals with systemic lupus erythematosus (SLE).^23^ NETosis has also been linked to cancer progression and metastasis in several studies.^24^, ^25^ As a result, a better understanding of the mechanism of NETosis may lead to new approaches for treating NET‐induced diseases, and targeting the process and products of NETosis is critical for treating diseases linked with NETosis.

In this review, we highlighted the mechanism of NETosis, the different receptors that might cause NET development, the link between diseases and NETosis, and potential strategies for treating diseases by targeting NETs.

## 2 THE TWO KEY MECHANISMS OF NETosis

**FIGURE 1:**
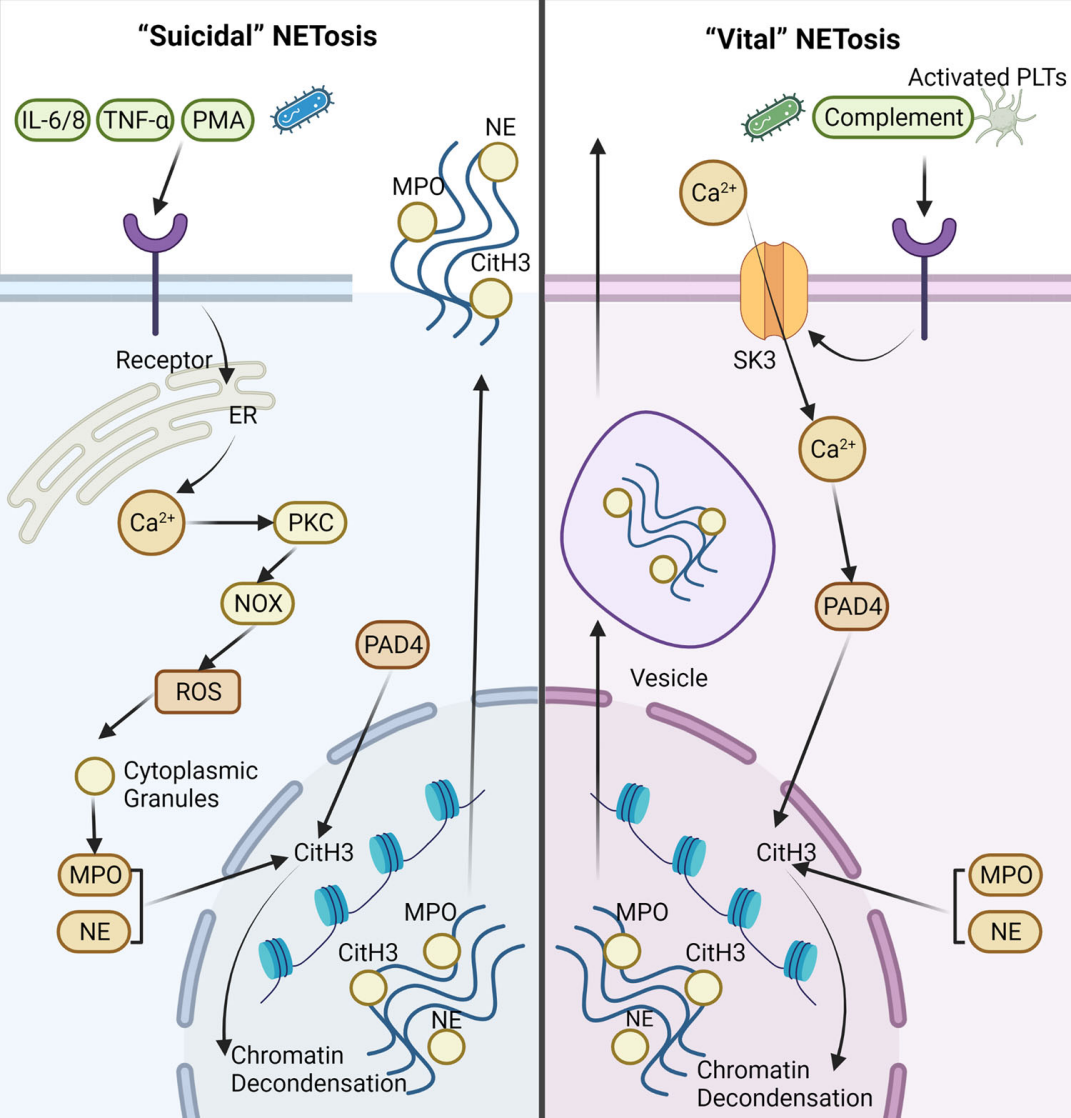
Two types of process of neutrophil extracellular trap (NET) formation (NETosis): “suicidal” and “vital.” Various materials, including pathogens, cytokines, and phorbol 12‐myristate 13‐acetate (PMA), can induce “suicidal” NETosis. Calcium ions can be released, leading to protein kinase C (PKC) activation when the receptors interact with these stimuli. Subsequently, the levels of reactive oxygen species (ROS) are elevated by activated NADPH oxidase complex (NOX), which promotes the degradation of cytoplasmic granules containing myeloperoxidase (MPO) and neutrophil elastase (NE). Along with peptidyl arginine deiminase 4 (PAD4), NE, and MPO induce the citrullination of histone H3 (CitH3), further resulting in chromatin decondensation. Finally, NETs are formed and released into the extracellular space. Another kind of NETosis is called “vital” NETosis, which can be activated by platelets (PLTs), microorganisms, and complement proteins. After the activation of neutrophils, calcium ions transfer into neutrophils via SK3. The rise in Ca^2+^ activates the PAD4 enzyme, leading to CitH3 and chromatin decondensation. Finally, the NET is sent out of the neutrophil by vesicles. Abbreviations: IL, interleukin; TNF‐α, tumor necrosis factor‐alpha

Current research evidence shows that NETosis is classified into two categories: (i) “suicidal” NETosis, which occurs in the presence of NADPH oxidase activity, and (ii) “vital” NETosis, which occurs without NADPH oxidase activity^10^ (Figure 1).

The “suicidal” NETosis was discovered following the initial stimulation with PMA.^26^ Activation of neutrophils is, in fact, a prerequisite. Stimuli might include PMA, cholesterol crystals, and (auto) antibodies.^27^ Calcium ions may be released from the endoplasmic reticulum when neutral surface receptors are engaged by these stimuli. Subsequently, PKC is activated as a result of the increase in calcium concentration. The NADPH oxidase complex (NOX) may then be assembled and activated.^28^ As a consequence, ROS such as O_2_, H_2_O_2_, HOCl, and others are produced.^29^ Regardless of its bactericidal effect, ROS plays a critical role in “suicidal” NETosis. For example, ROS may stimulate the degradation of cytoplasmic granules containing MPO and NE.^30^ Furthermore, with the help of ROS, NE can move into the nucleus and subsequently begin to cleave histones, resulting in chromatin decondensation.^31^ Meanwhile, the peptidyl arginine deiminase 4 (PAD4) enzyme is activated, promoting histone H3 citrullination, which is required for chromatin decondensation.^32^ NETs are formed by the combination of chromatin and granule proteins and are released into the extracellular space once the cell membrane ruptures. However, there is no doubt that neutrophils will die after this type of NETosis.

There are important molecules involved in the creation of NETs. Activation of PAD4 during NETosis is required for the rupture of cytoplasmic granules, chromatin decondensation, and the release of nuclear DNA into the cytoplasm.^33^ Sprenkeler et al.^34^ revealed that a complete and active actin polymerization process, as well as active myosin II, are critical for DNA release during NETosis. Cyclin‐dependent kinases (CDK) also play a key role in NET production; NETs are not produced until CDK4 and CDK6 are activated.^35^ Therefore, the rupture of the nuclear membrane may be intimately related to the cell cycle. Studies have shown that mitochondrial DNA (mtDNA), rather than nuclear DNA, may constitute the raw material of NETs in some diseases, including SLE and trauma.^36^, ^37^ Mitochondrial ROS (mitoROS) may play a role in Ca^2+^ influx. However, whether mitoROS is implicated is unclear.^28^ Furthermore, CO_2_ and HCO_3_^–^ levels may influence NETosis efficiency, and pH may alter the effectiveness of glycolysis in neutrophils regarding the “suicide” NETosis.^38^

A different type of NETosis is “vital” or NOX‐independent pathway. In contrast to the previously described “suicidal” NETosis, the “vital” NETosis takes approximately 30 min, while the “suicidal” NETosis lasts 3–4 h. Furthermore, the cause of these two forms of NETosis is also quite different. “Vital” NETosis can be stimulated by activated platelets, microorganisms, and complement proteins.^39^, ^40^ Calcium ions enter neutrophils after activation, which is regulated by small conductance potassium channel member three (SK3).^41^ The influx of Ca^2+^ activates PAD4 to aid in H3 citrullination. As a consequence, the electrostatic bond between histone and DNA weakens, causing chromatin decondensation.^42^, ^43^ Vesicles transport these chromatins, together with histones and granular proteins, out of the neutrophil.^44^, ^45^ The neutrophils are still alive after “vital” NETosis, and NOX is not needed during the process.

Research indicates that cleavage of the histone n‐terminal may be used to determine the type of NETosis; this is because NE cleaves the n‐terminal tails of core histones during “suicidal” NETosis but not “vital” NETosis.^46^ Nonetheless, more data about these two types of NETosis should be further explored.

## 3 THE RECEPTORS THAT MEDIATE NETosis

**FIGURE 2:**
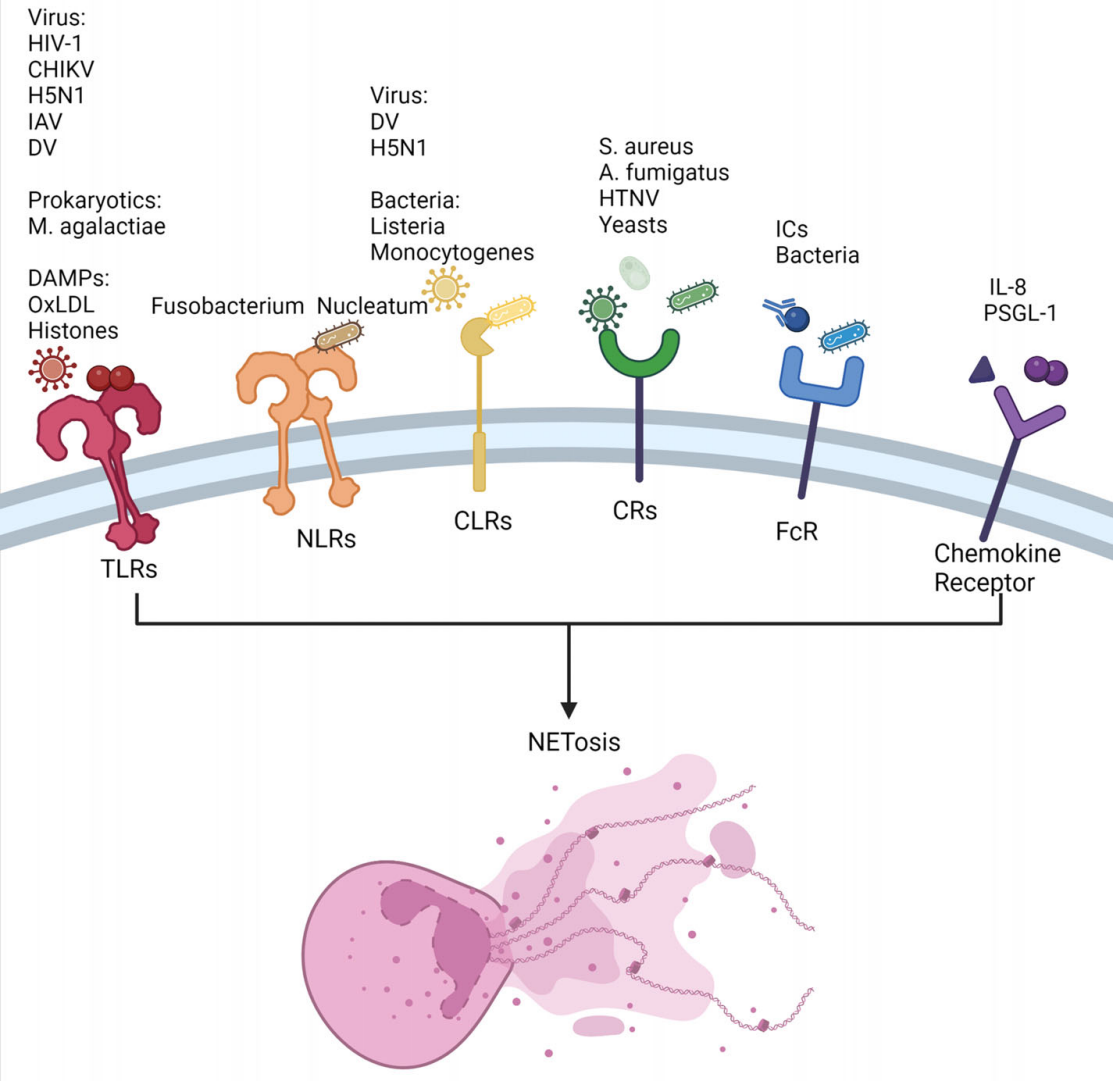
Receptors and corresponding stimuli related to process of neutrophil extracellular trap (NET) formation (NETosis). NETosis can be induced by multiple factors, including cytokines, immune complexes (ICs), oxidized low‐density lipoprotein (oxLDL), viruses, bacteria, yeasts, etc. In response to the different triggers, neutrophils can be activated by different receptors, including toll‐like receptors (TLRs), nucleotide‐binding oligomerization domain‐like receptors (NLRs), C‐type lectin receptors (CLRs), complement receptors (CRs), FcR, and chemokine receptors, and induce NETosis through different cellular signals. Abbreviations: CHIKV, chikungunya virus; DAMP, death‐associated molecular pattern; DV, dengue virus; HIV‐1, immunodeficiency virus‐1; HTNV, Hantaan virus; IAV, H5N1 influenza virus; IL, interleukin; PSGL, P‐selectin glycoprotein ligand

Receptors are biological macromolecules that are able to bind to hormones, neurotransmitters, drugs, or intracellular signal molecules and alter cell functions. Cell surface receptors and intracellular receptors are classified based on their location in cells. Neutrophils, which are at the forefront of recognizing and killing pathogens, have a variety of receptors that are vital to the human immune system. Neutrophils may exert immunological effects such as degranulation, ROS generation, and NET formation by recognizing certain ligands. The activation of neutrophil receptors is critical for both innate and adaptive immunity, and a vast number of receptors are also involved in the NETosis process (Figure 2). Dysregulated NETosis is related to the pathogenesis of various diseases. Therefore, it is vital to determine the relationship between receptors and NETosis.

### 3.1 Pattern recognition receptors

Pattern recognition receptors (PRRs) are a kind of recognition molecule that are mostly expressed on the surface and endosome of cells and can recognize one or more pathogen‐related molecular patterns (PAMPs). There are several types of PRRs found on neutrophils, including toll‐like receptors (TLRs), C‐type lectin receptors (CLRs), RIG‐I‐like receptors, absent in melanoma 2‐like receptors and nucleotide‐binding oligomerization domain‐like receptors (NLRs). Previous research has shown that TLRs, NLRs, and CLRs all play a role in the NETosis process.^28^

#### 3.1.1 Toll‐like receptors

TLRs are single transmembrane proteins that can identify microbial compounds with conserved structures. Current research has revealed 11 family members of TLRs, with eight of them (TLR1, TLR2, TLR4, TLR5, TLR6, and TLR11) being present on the cell surface.^47^ Human neutrophils express all TLRs except TLR3.^48^ TLR activation allows neutrophils to initiate the process of cytokine synthesis, ROS generation, NET formation, and degranulation.^49^ Several TLRs have been revealed to play a role in NET development. For example, various viruses, such as respiratory syncytial virus,^50^ chikungunya virus,^51^ H5N1 influenza virus,^52^ and dengue virus (DV),^53^ can induce the release of NETs through TLRs. Moreover, human immunodeficiency virus‐1 can be eliminated by NETs via the interaction of viral nucleic acids and TLR7/8.^5^ In addition to viruses, other pathogens contribute to NET formation. Research shows that *Mycoplasma agalactiae*, one of the world's smallest prokaryotes, may induce NETosis via the TLR2 signaling pathway.^7^ In addition, death‐associated molecular patterns (DAMPs), including oxidized low‐density lipoprotein and histones, may activate TLRs, resulting in NET release.^54^ Moreover, Huang et al.^55^ found that high‐mobility group box 1 (HMGB1) and histone DAMPs can induce NETosis by activating PAD4 via the TLR4 and TLR9 signaling pathways in liver ischemia/reperfusion (I/R) injury.

#### 3.1.2 NOD‐like receptors

NLRs are cytoplasmic receptors that provide a second line of defense against pathogens. Not only PAMPs but also DAMPs are recognized by NLRs. Notably, NLR1 and NLR2 are two of the most well‐studied NLRs.

There have been a few investigations on NETosis through the NLR pathway. The first report was in 2019, whereby *Fusobacterium nucleatum* was found to activate neutrophils, resulting in NETosis via the NOD1 and NOD2 pathways.^56^ Furthermore, recent research found that nod‐like receptor protein 3 (NLRP3) is associated with NETosis and that inhibiting NLRP3 might limit NET formation.^57^ However, the mechanism remains unknown.

#### 3.1.3 C‐type lectin receptors

CLRs are one of the most important PRRs with a Ca^2+^‐dependent sugar recognition domain. Similar to other PRPs, CLRs can identify extracellular and intracellular pathogens and bind PAMPs to inhibit microbial growth and promote phagocytosis. Neutrophils express some CLRs, including C‐type lectin‐2 (CLEC‐2), myeloid inhibitory C‐type lectin, Dectin‐1, and C‐type lectin (Mincle).^58^

CLEC2 and C‐type lectin member 5A (CLEC5A) are involved in NET release against pathogens such as DV and H5N1.^52^, ^59^ Furthermore, CLEC5A can induce immune responses, including NETosis and ROS production, against *Listeria monocytogenes* in mice.^60^ Mincle, another type of CLR, can induce NET release via an ROS‐independent pathway.^61^ In addition to stimulating NETs, CLRs can stop NET formation. Neutrophils may detect the size of the pathogen via a CLR called Dectin‐1, which helps them decide whether or not to release NET. Dectin‐1 can inhibit NE transport to prevent NET formation if the pathogen is extremely small.^19^

### 3.2 Complement receptors

Complement receptors (CRs) on the cell surface can mediate several biological effects, such as phagocytosis, immune regulation, adhesion, clearance of immune complexes, and inflammation, by engaging with the active fragments generated during complement activation. There are several CRs expressed on lymphoid and myeloid cells, and some of them, including CR4, CR3 (Mac‐1 or CD11b/CD18), CR1 (CD35), and CR5, are linked to NETosis.

Bacteria may activate neutrophils, resulting in the production of NETs through CRs. *Staphylococcus aureus* and *Aspergillus fumigatus*, for example, can cause NETosis via CR3 interaction.^62^, ^63^ Viruses may also activate neutrophils through CRs. For example, the Hantaan virus can cause NETosis through the ROS‐dependent pathway, mediated by CR3 and CR4.^64^ In addition, yeasts may activate neutrophils, resulting in NET production, which is dependent on CD18.^65^ Furthermore, C5a receptor‐1 (C5aR1) overexpression is thought to be linked to MPO–DNA, which is used as a NET marker in some patients with stable coronary artery disease.^66^

### 3.3 Fc receptors

Fc receptors are receptors for the C‐terminal of immunoglobulin (Ig) Fc. After Ig binds to an antigen, the Fc segment of the antibody becomes allosteric and binds to the Fc receptor on the cell membrane, resulting in various biological effects. As a result, Fc receptors are vital in regulating immune function and regulation, and each type of Ig has a corresponding Fc receptor. Human neutrophils express two Fc to recognize IgG molecules, including FcgRIIa (CD32a) and FcgRIIIb (CD16b).^67^

Previous findings indicated that immune complexes (ICs) could induce NET formation. Two separate studies showed that the mechanism of NETosis mediated by FcRs was significantly distinct. In one of the studies, FcgRIIa‐induced NETosis after FcgRIIIb promoted ICs endocytosis.^68^ However, another study found the opposite results.^69^ Furthermore, FcRs are potentially involved in NET formation to protect against bacterial invasion.^69^, ^70^ For example, studies have shown that hypervirulent *Klebsiella pneumoniae* (hvKp) and *S. aureus* can activate FcRs, leading to NET formation.^62^, ^71^

### 3.4 Chemokine receptors

Chemokine receptors, expressed on the cell membranes of immune cells and endothelial cells, are a type of G protein‐coupled receptor that mediates chemokine activity. Chemokine receptors can recruit neutrophils to sites of infection, trauma, and abnormal proliferation through chemokine interactions. CRs are divided into four main subfamilies: CXCR, CCR, CX3CR, and XCR. Among them, CXCR1, CXCR2, CXCR4, and CXCR7 are thought to be associated with NETosis.

Teijeira et al.^72^ found that CXCR1 and CXCR2 agonists were the main inducers of cancer‐promoted NETosis. Moreover, researchers have indicated that NET formation via the CXCR1/2 pathway is a therapeutic target in sepsis.^73^ CXCR2 has been linked to the progression of atherosclerosis (AS) and diffuse large B‐cell lymphoma through NET release.^74^, ^75^ The process of NETosis is activated via Src kinase, extracellular signal‐regulated kinase, and p38 mitogen‐activated protein kinase signaling following the interaction of interleukin‐8 (IL‐8) and CXCR2; Ca^2+^ is potentially involved in this process.^76^ In addition, CXCR2 can recruit neutrophils in the presence of P‐selectin glycoprotein ligand‐1, resulting in NETosis.^77^ CXCR4 is thought to play a significant role in NETosis in patients with severe malaria.^78^ Ngamsri et al.^79^ found that CXCR4 and CXCR7 inhibitors could inhibit NETosis and the production of platelet–neutrophil complexes, a biomarker for peritonitis and peritonitis‐associated sepsis.

## 4 THE DISEASES ASSOCIATED WITH NETosis

### 4.1 Sepsis

**FIGURE 3:**
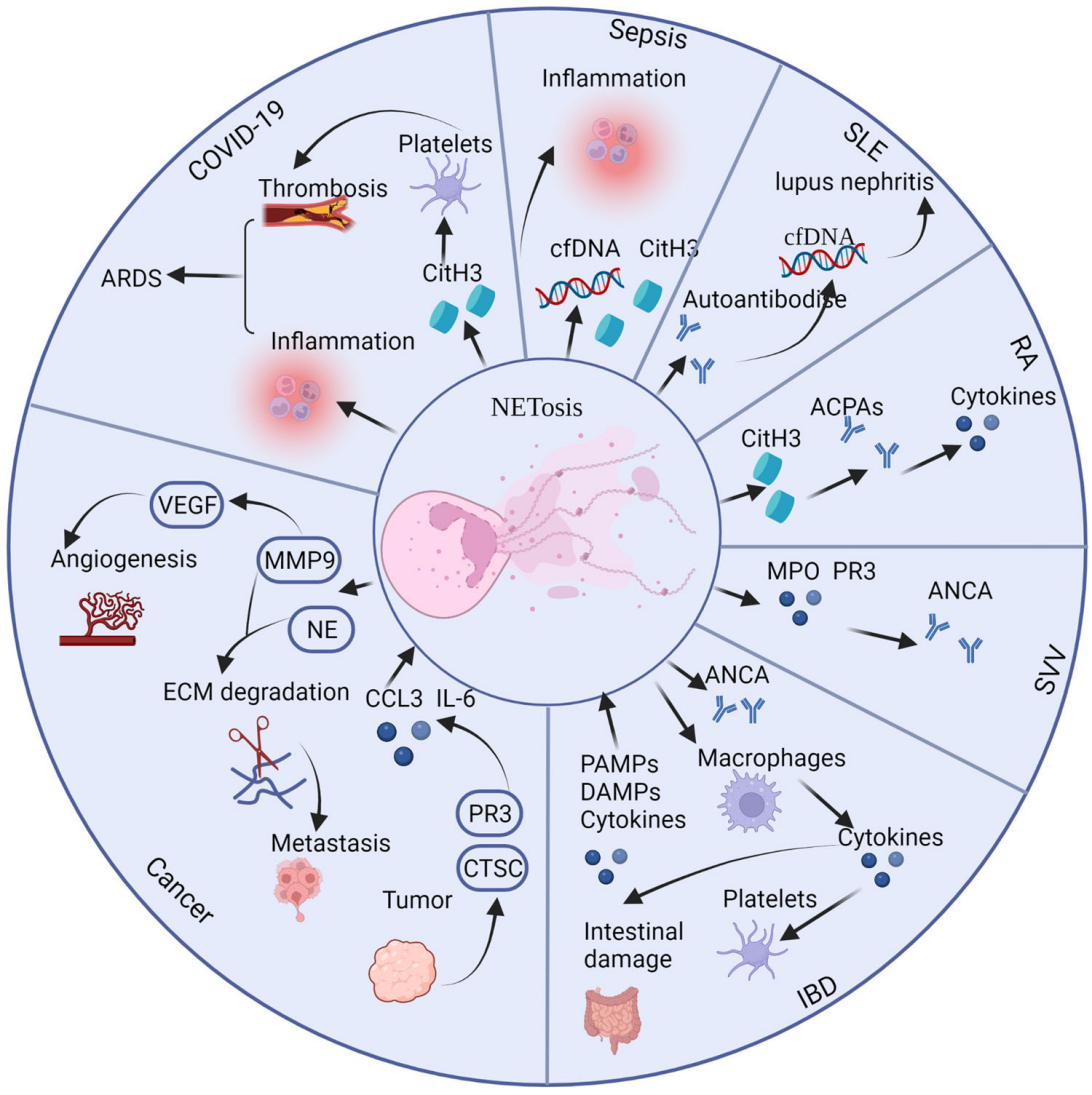
The roles of process of neutrophil extracellular trap (NET) formation (NETosis) in multiple diseases. NETs are involved in the pathogenesis and progression of various diseases, such as sepsis, systemic lupus erythematosus (SLE), rheumatoid arthritis (RA), small‐vessel vasculitis (SVV), inflammatory bowel disease (IBD), cancer, and COVID‐19. Components of NETs may act as autoantigens, leading to inflammation and autoimmune diseases. In addition, some diseases aggravate NETosis and cause a vicious circle. Abbreviations: ACPA, anti‐citrullinated protein antibody; ANCA, anti‐neutrophil cytoplasmic antibody; ARDS, acute respiratory distress syndrome; CitH3, citrullination of histone H3; cfDNA, cell‐free DNA; CTSC, tumor‐secreted protease cathepsin C; DAMP, death‐associated molecular pattern; IL, interleukin; MMP9, matrix metallopeptidase 9; MPO, myeloperoxidase; NE, neutrophil elastase; PAMP, pathogen‐related molecular pattern; PR3, proteinase 3; VEGF, vascular endothelial growth factor

Reports indicate that NETosis promotes sepsis, which is caused by bacteria and other pathogenic microorganisms invading the body and causing a systemic inflammatory response and even multiorgan failure. The release of inflammatory mediators is augmented by NETosis products such as cfDNA (Figure 3).^80^ Research has indicated that extracellular cold‐inducible RNA‐binding protein activated receptor on myeloid cells‐1 (TREM‐1) leading to the production of neutrophils with intercellular adhesion molecule‐1, which is involved in Rho GTPase to promote NETosis in sepsis.^81^ Extracellular histones produced by NETosis may act as DAMPs, causing inflammation and organ damage.^82^ Vitamin C might be used to treat sepsis patients by inhibiting the production of NETs.^83^ Studies show that vitamin C can reduce the level of cfDNA^83^ as well as block the activation of IκB kinase and NFκB.^84^ Nagaoka et al.^85^ discovered that LL37 can be used to treat sepsis by modulating NETosis. Moreover, a study showed that PAD2 protein levels are elevated in sepsis patients. They found that PAD2 inhibitors can reduce NET formation and ultimately increase the survival and organ function of sepsis patients.^86^

### 4.2 Systemic lupus erythematosus

SLE is an autoimmune disease that affects various organs and is caused by immunological complexes and excessive B‐cell proliferation. It is noteworthy that NETosis is strongly linked to the pathogenesis of SLE, and NET products are an important source of SLE autoantigens.^23^, ^87^ Under normal conditions, NETs might be degraded in serum. However, patients with SLE have autoantibodies against histones and cfDNA, which protects NETs from degradation.^88^ Furthermore, insufficient clearance of dead cells caused by NETosis may result in increased production of autoantibodies.^89^ The presence of DNase1 inhibitors/antibodies or the arrest of the link between DNase1 and NETs by complement component C1q or antibodies of NETs may explain the inefficient degradation of NETs.^88^, ^90^ The levels of circulating cfDNA are elevated because of the inefficient clearance and excessive production of NETs, which may contribute to the progression of lupus nephritis.^91^

Citrullinated histone from NETosis has been shown to play an important role in SLE pathogenesis.^92^ Autoantibodies in the serum of SLE patients preferentially combine with citrullinated core histones in NETs rather than non‐deiminated chromatin.^92^ Citrullination of histone H1 creates novel autoantibody epitopes that may stimulate B cells to make autoantibodies. Moreover, research shows that NETs with IL‐17A and tissue factor promote thromboinflammation and fibrosis in SLE via the REDD1/autophagy pathway.^93^ Cl‐amidine has been shown to inhibit the development of NETs in SLE diseases.^94^, ^95^ Additionally, acetylation and methylation of histones are associated with SLE. Posttranslational modifications of NETs from SLE‐derived neutrophils have a higher level of histone acetylation and methylation than NETs from healthy donors.^96^

It is generally known that the activation of autoreactive B cells is one of the SLE biomarkers. LL37–DNA complex, production of NETs, can activate polyclonal B cells via TLR9. In this view, the number of self‐reactive memory B cells against LL37 increases via an antigen‐dependent pathway.^97^

### 4.3 Rheumatoid arthritis

Rheumatoid arthritis (RA) is a chronic autoimmune disease that causes nonspecific inflammation of peripheral joints, resulting in the destruction of intra‐articular cartilage and bone, joint dysfunction, and even disability.

Numerous studies have shown that NETosis participates in RA progression. The synovial fluid of RA patients contains neutrophils, which may generate NETs.^98^, ^99^ The debris of NETs, including elastase, citrulline histone H3, and MPO, have been discovered in the synovial fluid and serum of RA patients.^46^, ^100^ Peptidylarginine deaminases (PAD) play a significant role in the production of NETs.^32^ Two PAD enzymes, PAD2 and PAD4, are required for the citrullination of several proteins in NETs, including actin, histone H3, α‐enolase, and vimentin, in RA.^101^ In this way, B cells can produce anti‐citrullinated protein antibodies (ACPAs) with the support of T cells.^98^, ^102^ ACPAs are found in more than two‐thirds of the sera of RA patients and are more specific than rheumatoid factors.^103^ Moreover, anticyclic citrullinated peptide antibody has emerged as a key biomarker for RA.^103^ These citrullinated autoantigens are potent inducers of proinflammatory cytokine release and NET formation. Research evidence shows that specific cytokines such as tumor necrosis factor‐alpha (TNF‐α), IL‐17A, and IL‐8 can induce NETosis in RA neutrophils.^104^ Therefore, NETosis may be a source of autoantigens, and the ensuing ACPAs can cause the development of NETs and a subsequent inflammatory response. Moreover, research indicates that quercetin inhibits NETosis by regulating autophagy in RA mice. Therefore, quercetin might be a potential therapy for RA by suppressing neutrophil activities.^105^ Navrátilová et al.^106^ found that NETosis can induce S100A11 (calgizzarin) release, which is related to the pathogenesis of RA.

### 4.4 Small‐vessel vasculitis

Small‐vessel vasculitis (SVV) is an autoimmune disease characterized by the appearance of anti‐neutrophil cytoplasmic antibodies (ANCAs) against MPO and PR3.^107^, ^108^ Kessenbrock et al.^108^ revealed increased levels of NETs in the blood of SVV patients. Immunofluorescence can reveal NETs in the necrotizing lesions of SVV. Furthermore, IgG antibodies in the sera of SVV patients were more likely to stimulate NET production in vitro than those in the control group. As previously noted in the section on SLE, the activity of DNAse1 is similarly decreased, resulting in NET accumulation in SVV patients.^109^ In addition, Wang et al.^110^ indicated that NETs can activate the alternative complement pathway participating in the pathogenesis of ANCA‐associated vasculitis (AAV). In addition, α‐PR3 and α‐MPO ANCAs can trigger NETosis, resulting in a vicious circle.^108^ The dysregulated release of NETs may induce endothelial cell damage because of their cytotoxic effect.^107^, ^108^ However, several investigations have shown that ANCA IgG may trigger NETosis regardless of ANCA level,^111^ and circulating NET levels cannot be used to diagnose SVV patients.^112^

### 4.5 Inflammatory bowel disease

Inflammatory bowel diseases (IBDs), which include ulcerative colitis (UC) and Crohn's disease (CD), are a category of gastrointestinal diseases characterized by chronic inflammation. Clinical evidence shows that IBD symptoms include severe diarrhea, fluid loss, abdominal pain, and bleeding.^113^, ^114^ Although the origin of IBD is unknown, new research suggests that neutrophils play a significant role in IBD pathogenesis, and neutrophil condensation in the intestinal mucosa is positively correlated with the severity of UC and CD.^115^, ^116^ ANCAs are a key biomarker for IBD, and ANCAs can target neutrophil proteins produced during NETosis.^117^

Research evidence indicates that NETosis may exert different effects on UC and CD. A recent study found that NETosis is more prevalent in CD and UC patients.^118^ However, the formation of NETs has been previously found to be solely associated with UC and not with CD, suggesting that the pathogenesis of UC is related to innate immune system activation.^119^

Multiple inducers, including PAMPs, DAMPs, and cytokines, that can activate NETosis in IBD patients have been reported. Previous research by Dinallo et al.^120^ demonstrated that stimulating lipopolysaccharide (LPS) could increase NET production in UC patients. Cytokines, such as TNF‐α, IL‐1β, and IL‐6, were also detected in IBD.^121^

Proteins from NETs cause tissue damage in IBD patients. These proteins are linked to IBD pathology and produce inflammatory responses, extracellular matrix (ECM) degradation, and other severe outcomes. NETs may induce macrophages to release proinflammatory cytokines such as TNF‐α, IL‐6, and monocyte chemotactic protein‐1, which leads to platelet activation and intestinal damage.^120^ PAD, a major NETosis product, plays a role in the pathogenesis of IBD by increasing proinflammatory cytokine levels in conjunction with MPO and decreasing the anti‐inflammatory cytokine IL‐10.^122^

### 4.6 Cancer

Cancer is one of the deadliest diseases, with unrestricted cell proliferation and invasive growth. Recent research shows that NETosis is closely associated with cancer progression and metastasis.^24^ Furthermore, circulating levels of NET components, including cfDNA, NE–DNA, MPO–DNA, and CitH3, are useful biomarkers for several cancers. However, the relationships between their levels and cancer diagnosis and/or progression are not fully understood.

Tumor cells and their microenvironment may cause NETosis. Probably, NET formation is caused by the hypoxic environment generated by solid tumors with high levels of hypoxia‐inducible factor‐1α.^123^ Xiao et al.^124^ found that the tumor‐secreted protease cathepsin C may activate neutrophil membrane‐bound PR3, resulting in an increase in CCL3 and IL‐6 and recruitment of neutrophils. Furthermore, Leal et al.^125^ demonstrated that tumor‐derived exosomes from cancer patients in prethrombotic stages may cause NETosis. Tumor cells can also directly induce NETosis by secreting granulocyte‐colony stimulating factor.^126^

NETs potentially promote tumor progression and metastasis. Experiments have shown that NETs promote tumor progression by enhancing mitochondrial activity in tumor cells to provide more energy.^127^ Recent research indicates that NE, which is released during NETosis, can promote cancer progression. Houghton et al.^128^ found that NE‐defective mouse models of lung adenocarcinoma exhibit decreased tumor burden and mortality when compared to controls. Matrix metallopeptidase 9 (MMP9), a NETosis product, may promote cancer progression by increasing angiogenesis via the production of vascular endothelial growth factor.^129^ Moreover, NE and MMP9 can cleave laminin, which is a significant component of the ECM,^130^ and ECM degradation to the damaged basement membrane is a prerequisite for tumor cell invasion and metastasis.^131^ Tohme et al.^123^ discovered that NETs derived from mouse neutrophils may induce the production of HMGB1, which activates tumor cells through the TLR9 pathway. Ortiz‐Espinosa et al.^132^ found that C5a may trigger polymorphonuclear myeloid‐derived suppressor cells (PMN‐MDSCs) to release NETs, hence aiding cancer cell migration and metastasis. Metastasis could be minimized in a mouse lung metastasis model by blocking C5a, C5aR1, or NETosis.

Tumor cells that infiltrate the peripheral blood stream are known as circulating tumor cells (CTCs). CTCs may persist and cause metastasis in the absence of the immune system and blood flow shear forces.^133^ Platelets produced by NETosis may protect CTCs from immune cells due to the trapping properties of NETs.^134^ Experimental evidence shows that NETs produced by surgical stress‐activated platelets may enhance CTC trapping and distant metastasis. Moreover, by depleting or blocking platelets, metastasis can be minimized or stopped.^135^ Thus, disrupting the interaction between platelets, tumor cells, and NETs might be a key target for cancer treatment.^131^ Moreover, NETs can increase tumor cell extravasation by entrapping or attaching them to capillaries. A study demonstrated that NETs may adhere to CTCs via β1‐integrin, resulting in extravasation.^136^

### 4.7 COVID‐19 and acute respiratory distress syndrome

The COVID‐19 virus emerged around the end of 2019 and continues to spread and mutate fast, resulting in a new wave of the pandemic in regions throughout the world. COVID‐19 patients experience flu‐like symptoms and viral pneumonia, which can develop into acute respiratory distress syndrome (ARDS) or potentially multiple organ failure.^137^

Recent research identified higher levels of NETs in the tracheal aspirate and lung tissues of patients with severe COVID‐19,^138^ and NET components may elicit an inflammatory response and vascular microthrombosis, resulting in ARDS.^139^ Clinical studies have shown that individuals with COVID‐19 have increased amounts of cfDNA, MPO–DNA complexes, and CitH3, all of which are important components of NETs.^140^ In vitro, serum samples from COVID‐19 patients may stimulate NET formation in control neutrophils. Moreover, cfDNA is linked to acute‐phase reactants and lactate dehydrogenase, as well as neutrophil count, while CitH3 is linked to platelet levels, supporting a function for NETosis in thrombosis.^140^ Notably, SARS‐CoV‐2 can directly cause spontaneous NET release in vitro.^141^, ^142^

Several studies have demonstrated a positive correlation between the presence of NETs and COVID‐19 severity. Zuo et al.^140^ reported higher levels of cfDNA and MPO–DNA in patients treated with specific care and mechanical ventilation than in those breathing room air. Lower levels of PaO_2_:FiO_2_ ratio suggest increasing respiratory failure as an essential indicator of the severity of respiratory failure. Experimental evidence shows that the PaO_2_:FiO_2_ ratio correlates inversely with NET levels. Furthermore, the SOFA score, which is a clinical disease severity index, is linked to NET levels.^138^

Furthermore, NETs can trap and eliminate pathogens to protect the host against viral infection. However, we found that histones, a major component of NETs, could enhance SARS‐CoV‐2 infection.^142^ Mounting evidence shows that NETosis is associated with thrombosis, which is a significant predictor of disease severity in COVID‐19 patients.^143^ In COVID‐19 patients, researchers discovered inflammatory microvascular thrombosis with NET‐associated fibrin and platelets in the heart, lung, and kidney; several neutrophil–platelet aggregates in the patients suffered from COVID‐19.^144^

### 4.8 Other diseases

Acute renal injury (AKI) is a group of clinical syndromes that refers to a sudden and continuous sudden decline in renal function. Research showed that NET release and tubular necrosis caused histone and cytokine release, promoting kidney injury.^145^ Henry et al.^146^ indicated that intravascular NETosis was related to the pathogenesis of COVID‐19‐associated AKI and microthrombosis.

Several studies have suggested that NETosis is associated with pancreatitis. Leppkes et al.^147^ found that NETosis promotes pancreatitis by ductal occlusion. Some special components in pancreatic juice, including calcium carbonate crystals and bicarbonate ions, lead to aggregated NET formation.

Gout is a common type of arthritis characterized by the precipitation of monosodium urate (MSU) crystals in the peripheral joints. Chatfield et al.^148^ found NETs in the fluid from acutely inflamed joints in gout patients. In addition, the uninflamed tophi were coated with NETs in patients with gout. Moreover, a recent study indicated that a decrease in GPR105, which is highly expressed in neutrophils and sensitive to MSU, can prevent NETosis and induce apoptosis. Therefore, targeting GPR105 might be a possible therapy for acute gouty arthritis.^149^

To cure multiple NET‐associated diseases, further research on NETosis is needed.

## 5 THERAPEUTIC STRATEGIES FOR TARGETING NETosis

**FIGURE 4:**
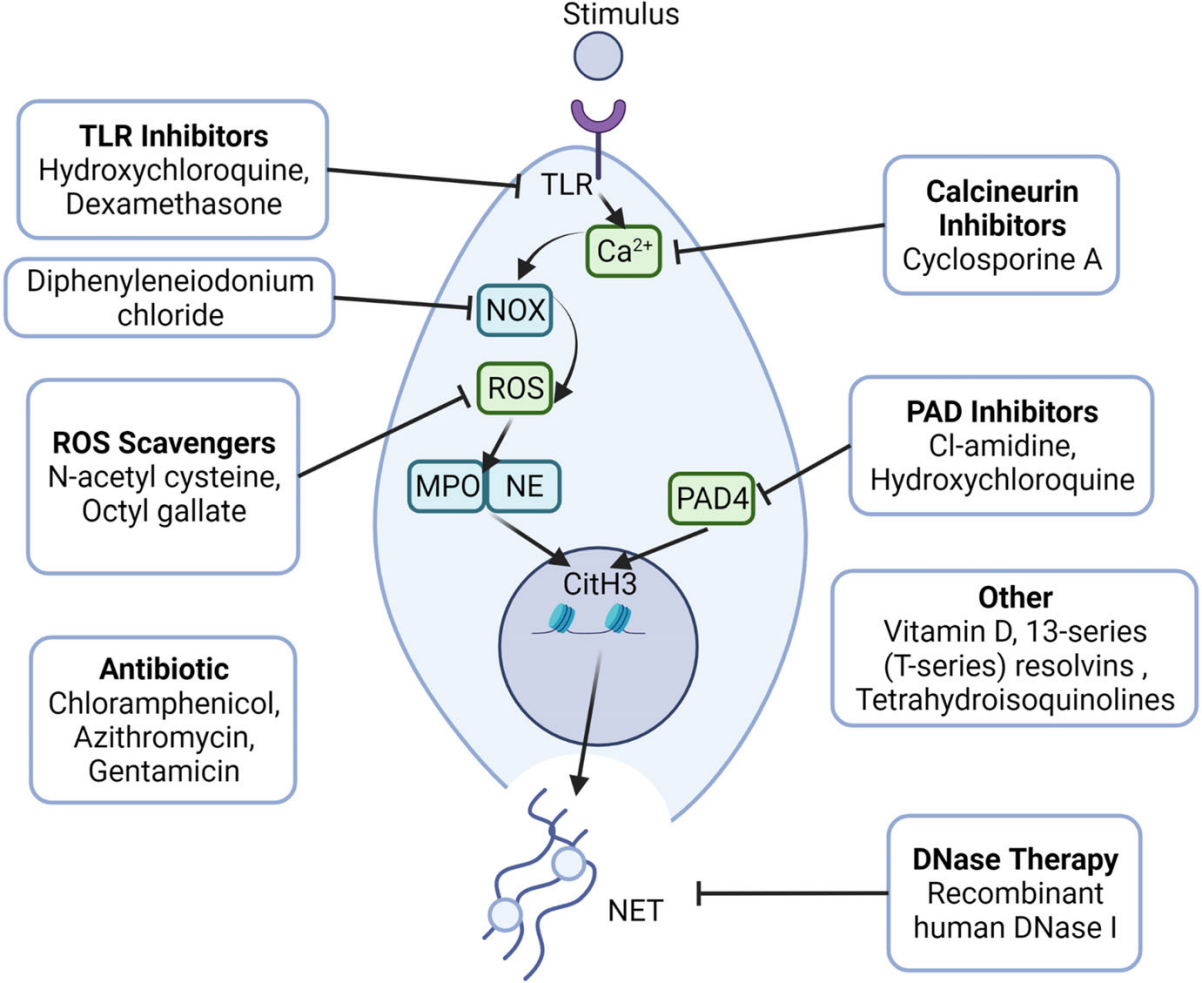
Therapeutic strategies targeting neutrophil extracellular trap (NET) formation. Regulating uncontrolled process of NET formation (NETosis) could be a promising strategy for the treatment of NET‐related diseases. DNase and multiple inhibitors can be utilized for targeting the critical steps and products of NETs. Abbreviations: CitH3, citrullination of histone H3; MPO, myeloperoxidase; NE, neutrophil elastase; NOX, NADPH oxidase complex; PAD4, peptidyl arginine deiminase 4; ROS, reactive oxygen species; TLR, toll‐like receptor

As previously stated, dysregulated NETosis is associated with the pathogenesis of various diseases. Therefore, regulating uncontrolled NETosis might be a viable strategy for treating NET‐associated diseases (Figure 4; Table 1).

**TABLE 1:** Potential anti‐neutrophil extracellular trap (NET) therapeutics and mechanism of action

Anti‐NET therapeutics	Pharmacological compounds	Phase	Target	Mechanism of action	Disease	Trial number
TLR inhibitors	Dexamethasone	Phase 4	TLR2 and TLR4	Regulate TLR2 and TLR4 without affecting ROS production	COVID‐19	NCT04707534
Calcineurin inhibitors	Cyclosporine A	Phase 2	Calcineurin	Inhibit calcineurin pathway, reduce activation of neutrophils	SLE	NCT00976300
ROS scavengers	N‐acetylcysteine	Phase 2	ROS	Reduce ROS formation, indirectly inhibit NET production	SLE	NCT00775476
	Methotrexate	Phase 3			SLE	NCT00470522
	Octyl gallate	Preclinical			SLE	
	Diphenyleneiodonium chloride	Preclinical				
PAD inhibitors	Cl‐amidine	Preclinical	PAD4	Decreasing atherosclerotic lesion Area and thrombosis	SLE, diabetes	
DNase	Recombinant human DNase	Phase 1	NET‐derived DNA	Degradation of NETs	COVID‐19	NCT04409925
Anti‐inflammatory/immunomodulatory	Azithromycin	Phase 3	Cytokines	Affect the activation and migration of neutrophils	Sepsis	NCT03199547
	Chloramphenicol	Preclinical				
	Gentamicin	Phase 4			Sepsis	NCT02898961
Other	Vitamin D	Phase 2		Reduces endothelial damage and cell apoptosis	SLE	NCT01709474
	13‐Series (T‐series) resolvins	Preclinical	Macrophage	Decrease NET release and promote NET clearance		
	Tetrahydroisoquinolines	Preclinical	Neutrophils	Reduce NETosis without influencing neutrophil activity		

Abbreviations: NETosis, process of NET formation; PAD4, peptidyl arginine deiminase 4; ROS, reactive oxygen species; SLE, systemic lupus erythematosus; TLR, toll‐like receptor.

*Source*: Clinical Registration website https://www.clinicaltrials.gov/ct2/home.

### 5.1 Chlor‐amidine

Chlor‐amidine (Cl‐amidine) inhibits protein‐arginine deiminase irreversibly by covalent modification of the active site of the enzymes.^150^ Experimental results demonstrated that 11 weeks of daily Cl‐amidine injections as a pharmacological inhibitor of PAD4 may reduce thrombosis and atherosclerotic lesion area via NET formation in the mouse model of AS. Furthermore, in a mouse model of SLE, Cl‐amidine may protect mice against NET‐induced outcomes such as kidney injury, endothelial dysfunction, and vascular damage.^151^ However, Gordon et al.^152^ found that PAD inhibitors did not alleviate the end‐organ damage features of proteinuria, showing that inducers other than NETosis cause autoimmune disease.

### 5.2 Hydroxychloroquine

Hydroxychloroquine (HDQ) is an antimalarial drug used for the treatment of malaria as well as other diseases, including SLE and RA.^153^, ^154^, ^155^ Studies have shown that HDQ can reduce NETosis by inhibiting TLR9 and suppressing the expression of PAD4 and Rac2.^156^, ^157^ As an immunomodulator, HDQ regulates cytokine production by inhibiting costimulatory molecules.^158^, ^159^ As an immunomodulator, HDQ regulates cytokine production by inhibiting costimulatory molecules.^160^, ^161^, ^162^ Zhang et al.^156^ found that HDQ inhibited NET formation in a mouse model of hepatic I/R injury. Moreover, HDQ was reported to prevent neutrophils from absorbing tumor‐derived extracellular vesicles, which participate in NET formation and intercellular transport.^163^, ^164^ In contrast, several studies reported that HDQ did not influence the expression of NE, PAD4, and MPO.^165^

### 5.3 Diphenyleneiodonium chloride

As a hypoglycemic agent, diphenyleneiodonium chloride (DPI) can suppress gluconeogenesis and respiration by inhibiting numerous enzymes, including NOX, NADPH cytochrome P450 oxidoreductase, NO synthase, xanthine oxidase, and cholinest erase.^166^, ^167^ It has been shown that DPI binds the heme group of NADPH oxidase to reduce ROS production and NADPH oxidase.^168^ DPI can also reduce the release of extracellular DNA during NET formation. However, the effects of DPI were distinguishing when it was used before or together with PMA stimulation.^169^

### 5.4 N‐acetylcysteine

N‐acetylcysteine (NAC), also called acetylcysteine, is a mucus lysine used for the treatment of chronic obstructive pulmonary diseases, bronchiectasis, and acetaminophen overdose.^170^, ^171^ It also exerts antioxidant effects in multiple diseases associated with ROS.^172^, ^173^ A previous study found that NAC suppressed ROS production to reduce NETosis in a dose‐dependent manner. However, NAC cannot reduce the formation of NETs in the presence of hydrogen peroxide, suggesting that NAC inhibits NETosis by regulating ROS production.^174^ Craver et al.^175^ showed that NAC reduced thrombus formation in vivo, which is similar to the effects on the nonreversible platelet inhibitor, aspirin. They also revealed that NAC reduced the formation of thrombin‐induced platelet–leukocyte aggregates in a mouse model with Janus kinase 2 mutation, which is common in patients with chronic hematologic malignancies (CHMs). Moreover, it decreased the release of NET in primary human neutrophils extracted from CHM patients or healthy individuals.^175^

### 5.5 Recombinant human DNase

Recombinant human DNase I (rhDNase I), which selectively cleaves DNA to degrade DNA in sputum, is a drug used for the treatment of bronchiectasis and lung abscess. DNase has multiple functions, including absorbing nucleotide nutrients, regulating biofilm formation, facilitating pathogen invasion, degrading DNA matrixes, and regulating immune functions.^176^, ^177^, ^178^ Studies showed that DNase degraded DNA‐nucleoprotein and ICs, thereby conferring therapeutic effects against lupus nephritis and SLE.^179^

Evidence from previous studies has shown that DNase regulates NETosis to reduce neutrophil infiltration and the inflammatory response.^180^, ^181^, ^182^ DNase also reduces NET formation in postischemic muscle,^183^ as well as the tumor volume in a mouse model when used together with other proteases, such as trypsin and papain.^184^ DNase therapy has also been reported to treat cystic fibrosis. Together with elastase, DNase degraded the DNA–protein complexes released from NETosis.^185^ However, treatment with DNase may trigger the release of cytotoxins from NETosis, thereby causing inflammation.^186^

### 5.6 Vitamin D

Vitamin D is a fat‐soluble vitamin also known as a cyclopentanoperhy drophenanthrene compound. Vitamin D can effectively treat numerous diseases, including rickets, chondrosis, osteoporosis, hypothyroidism, and psoriasis. In addition to increasing intestinal absorption of minerals, vitamin D can also activate the innate immune system and suppress the adaptive immune system.^187^, ^188^, ^189^, ^190^ Vitamin D deficiency is one of the most important symptoms of SLE patients. Handono et al.^191^ found that vitamin D reduced NET formation and the number of damaged endothelial cells. As an immunomodulator, vitamin D can be used as a therapy for SLE patients with hypo‐vitamin D to inhibit endothelial damage.

### 5.7 Antibiotic (azithromycin, chloramphenicol, gentamicin) and other drugs

Antibiotics are a class of secondary metabolites produced by microorganisms or higher animals and plants that are resistant to pathogens or other activities. In addition to their antibacterial function, antibiotics can also be used as immunomodulators, as they interact with multiple immune cells, such as neutrophils.^192^, ^193^ In a previous study, pretreatment of neutrophils with chloramphenicol and azithromycin reduced NET formation.^194^ Moreover, azithromycin treatment reduced respiratory burst in a concentration‐dependent manner. Manda‐Handzlik et al.^193^ showed that gentamicin suppressed NETosis, but cefotaxime did not. These results show that different antibiotics have different therapeutic effects.

Several other drugs that inhibit NETosis have been reported. For example, dexamethasone reduced NETosis by interacting with TLR2 and TLR4.^195^ It has been shown that cyclosporine A can inhibit the activity of neutrophils by blocking the calcineurin pathway, which regulates neutrophil activation.^196^ Chiang et al. reported that 13‐series (T‐series) resolvins can decrease NET release and promote NET clearance by macrophages via a cyclic adenosine monophosphate‐protein kinase A‐AMPK axis.^197^ Recently, Haute et al. found that octyl gallate reduced NETosis by inhibiting ROS production.^198^ In comparison, tetrahydroisoquinolines can reduce NETosis without influencing neutrophil activity, including the formation of ROS and neutrophil granular protein activity.^199^ However, the specific mechanism remains unknown.

## 6 CONCLUSION AND PERSPECTIVE

Numerous studies have revealed multiple effects of NETs on the host. Neutrophils are one of the most important immune cells for innate immunity. Depending on the type of stimuli, NETs are released via different pathways. Yipp et al.^200^ found a new method of NET formation in which neutrophils can release NETs within 10 min following *S. aureus* infection.

NETs released by neutrophils can trap or kill multiple pathogens, including bacteria, viruses, fungi, and protozoa. However, uncontrolled NETosis will cause tissue damage leading to inflammation or serious diseases. Dysregulated NETosis has been reported to induce or aggravate numerous diseases, including SLE, RA, SVV, sepsis, IBD, cancer, and ARDS. Although the details and mechanisms of multiple NET‐associated diseases are not fully understood, the levels of NET components have been shown to be potential biomarkers for the diagnosis and prognosis monitoring of these diseases. Targeting specific steps or products of NETosis can offer therapeutic benefits in NET‐associated diseases. Multiple drugs targeting different steps of NET formation have been reported, including Cl‐amidine, HDQ, DPI, NAC, rhDNase, vitamin D, antibiotics, and others. For example, several studies have shown that PAD enzymes can reduce NET formation and dampen the activity of diseases associated with NETosis, including diabetic wounds, colon cancer, AS, and RA.^95^, ^201^, ^202^, ^203^, ^204^ Moreover, NET inhibitors can be used in combination immunotherapies as adjuvants to improve the effectiveness of immune checkpoint blockers and other cancer drugs. Teijeira et al.^72^ indicated that combination therapy with checkpoint inhibitors and NET inhibitors can improve the antitumor ability of CD8^+^ T cells. However, these drugs are associated with negative effects on the host's immune system, such as increased susceptibility to infections and weakened immune systems. Combined therapy might be an effective approach to reduce these detrimental effects and improve their efficacy.^205^ Further studies are recommended to reveal the detailed connections between NETosis and NETosis‐related diseases and to identify strategies to effectively modulate dysregulated NETosis.

## CONFLICT OF INTEREST

All authors declare they have no conflicts of interest.

## AUTHOR CONTRIBUTIONS

L.Z. and M.W. have provided important guidance for this paper. J.H. drafted the manuscript and completed the illustrations and descriptions. W.H. provided the main writing ideas and further refined the article. All authors have read and approved the final manuscript.

## ETHICS STATEMENT

Not applicable.

## Data Availability

Not applicable.
